# Mitigation of Particle Spread During Mastoidectomy: A Systematic Review

**DOI:** 10.7759/cureus.19040

**Published:** 2021-10-25

**Authors:** Nicole M Favre, Kelcy M McIntyre, Cathleen C Kuo, Michele M Carr

**Affiliations:** 1 Otolaryngology, University at Buffalo, Jacobs School of Medicine and Biomedical Sciences, Buffalo, USA

**Keywords:** personal protective equipment, particle spread, mastoidectomy, coronavirus, covid-19

## Abstract

Our objective is to analyze the risk of particle spread through mastoidectomy during the COVID-19 pandemic with an aim to assess the tools used to mitigate the spread. A systematic review was conducted using PRISMA guidelines. Our search terms included: MASTOIDECTOMY + COVID-19 or MASTOIDECTOMY + SAR- CoV-2 or MASTOIDECTOMY + CORONAVIRUS. Studies consistent with the inclusion and exclusion criteria were included in the review. Of the 20 articles identified in the initial search, six met the inclusion criteria. The included articles were all experimental studies, with five studies using cadaver subjects and one study using live human subjects. Three studies measured droplet spread and three studies measured aerosolized particle spread. The maximum distance of particle spread ranged from 30 cm to 208 cm. Four studies assessed the use of a barrier system, with two using the OtoTent and two using a barrier drape. Two studies defined the microscope alone as a possible mitigatory tool. One study compared burr type and size to determine the effects on particle spread. During the coronavirus disease 2019 (COVID-19) pandemic, evaluation of tools to mitigate particle spread is imperative for the safety of the surgical team and the healthcare system at large. Barrier drapes, OtoTents and microscopes all have proven to mitigate particle spread; however, further research needs to be performed to compare their efficacy and develop a standard of safety.

## Introduction and background

On March 11, 2020, the World Health Organization declared the 2019 novel coronavirus (COVID-19) global pandemic [1] after multiple reports of pneumonia associated with a seafood market in China [2]. Since its original identification, COVID-19 has rapidly spread to 109 other countries [3]. SARS-CoV-2, the viral agent of COVID-19, has a transmission rate of R0: 2-3, meaning one infected person was expected to infect three others [4]. In addition, the virus was eventually reported to be transmissible by asymptomatic individuals, increasing the likelihood of community spread. Spread of the virus is thought to occur through person-to-person contact via respiratory particles within a range of approximately six feet [5]; however, SARS-CoV-2 can also be transmitted longer distances through airborne routes, particularly in enclosed, poorly ventilated spaces [6]. As of October 2021, there were approximately 44,000,000 confirmed cases with over 718,000 deaths related to COVID-19 in the United States (CDC).

As COVID-19 is spread through respiratory particles, there is a particular risk for otolaryngologists. During the early outbreak, reports described multi-person perioperative outbreak events which had occurred during endonasal skull-base surgery, raising concern for contagious aerosol spread [7]. An international study of 325 otolaryngologists, aged from 25 to 84 years, who were infected with COVID-19 found that 165 (54%) of cases were likely contracted during the surgeon’s clinical activity. There were 24 associated deaths. Six of the cases were found to be associated with the performance of aerosol-generating operations, including mastoidectomy, tracheostomy, epistaxis control, dacryocystorhinostomy, and translabyrinthine resection [8].

Surgical procedures which involve drilling or cutting of the bone are high risk for surgeons due to the spread of communicable diseases through aerosolized particles. Mastoidectomy, which is a common procedure performed for the treatment of chronic ear disease, is an example of a high-risk procedure. Previous research has indicated a spread of viral particles during mastoidectomy and found that there was an arc-like spread of bone particles upwards toward the surgeon while drilling [9].

In this systematic review, we analyzed the risk of COVID-19 infection through mastoidectomy with an aim to assess the tools used to mitigate the spread.

## Review

**Figure 1:**
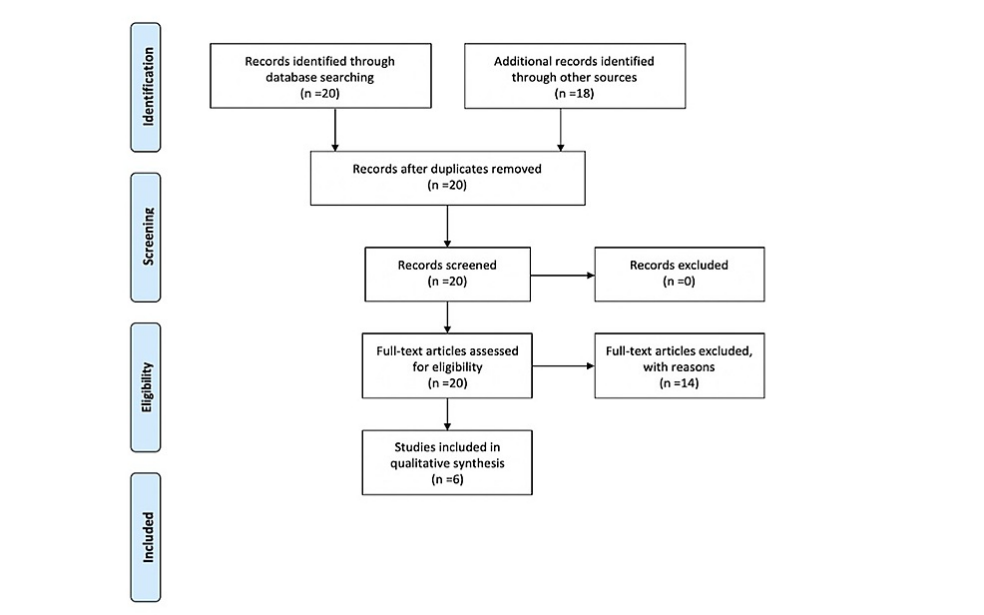
PRISMA flow diagram. PRISMA: Preferred Reporting Items for Systematic Reviews and Meta-Analyses.

We conducted a systematic review by searching PubMed and Google scholar without date, geographic location of study, or language restrictions (performed February 2021). PRISMA guidelines were followed (Figure 1) [10]. Our search terms included: MASTOIDECTOMY + COVID-19 or MASTOIDECTOMY + SAR- CoV-2 or MASTOIDECTOMY + CORONAVIRUS. 

To be eligible for inclusion in the analysis, the following criteria were required: study needed to assess mastoidectomy procedure, the study needed to assess the spread of particles OR tools used to mitigate particle spread OR use of personal protective equipment (PPE), and all articles were peer-reviewed.

Studies were excluded if: study did not assess mastoidectomy procedure, the study did not assess the spread of particles OR tools used to mitigate particle spread OR use of PPE.

Twenty novel articles were discovered by screening titles and abstracts of papers. For articles that appeared to meet the inclusion criteria, the full text was reviewed. Two reviewers independently screened the titles and abstracts of articles. Three reviewers reviewed and compiled the data from each article. Any disagreements were discussed among the three reviewers and agreed upon prior to final inclusion. We included year of publication, country of publication, type of study, subject, procedure completed, measurement of particle spread methods, measurement of particle spread results, tools used to mitigate spread, success of spread mitigation, PPE used, hindrance of PPE and suggested resolution of hindrance.

Results

Of the 20 articles identified in the initial search, six met the inclusion criteria. The included articles were all experimental studies, with five (83%) studies using cadaver subjects and one (17%) study using live human subjects. All six (100%) studies assessed mastoidectomy. Studies assessed cutting and diamond burrs from 3 mm to 6 mm in size. All mastoidectomies were performed at speeds up to 60,000 to 70,000 RPM. Three (50%) studies measured droplet particle spread and three (50%) studies measured aerosolized particle spread. Droplet particles are defined as virus-containing respiratory droplets which are > 5 micrometers, whereas aerosolized particles are <5 micrometers. The small size of aerosolized particles allows them to be suspended in the air longer and thus travel longer distances [11]. Among the studies we analyzed, the maximum distance of particle spread ranged from 30 cm to 208 cm (Table 1).

**Table 1:** Assessment of particle spread in mastoidectomy procedure. PSD: particle surface density; SA: surface area.

Author, year	Country	Type of study	Subject	Drill type and speed	Methods	Measurement of particle spread
Chari et al. 2021 [11]	United States	Experimental	Three Cadavers	Otologic drill with a compatible 6-mm round cutting burr and 5-mm diamond burr was used at 70,000 RPM	Number and size distribution of aerosol particle was quantified via optical particle size spectrometer	1-10 µm particle spread detected 30 cm from surgical site 10^5 µm/L PSD generated
Chen et al. 2020 [12]	United States	Experimental	Two Cadavers	Otologic drill with a compatible 6-mm round cutting burr was used at 70,000 RPM	Fluorescent aerosol particle surface density was quantified via segments of an octagonal test grid	100 µm - 4.6 mm particle spread detected 114 cm from the external auditory canal 0.036 to 4.0 particles/cm^2 ^PSD generated
Cottrell et al. 2020 [13]	Canada	Experimental	Human	Otologic drill with 6-mm cutting burr at speeds up to 70,000 RPM	Fluorescent aerosol dispersion characteristics were analyzed	Particle spread detected 173 cm from the ear (PSD not specified)
Lawrence et al. 2020 [14]	United Kingdom	Experimental	One Cadaver	Otologic drill at speeds up to 60,000 RPM (Burr type and size not specified)	Fluorescent droplet spread was assessed by a ‘black light’ in a darkened operating theater, and the results photographically documented	Particle spread detected 200 cm from the drilling site (PSD not specified)
Markey et al. 2021 [15]	United Kingdom	Experimental	Plastic Temporal Bone	Otologic drill with 3 to 5-mm cutting or diamond burr at speeds up to 60,000 RPM	Fluorescent droplet splatter was measured using a segmented experimental floor plan	Particle spread detected 208 cm in all directions using the 5 mm cutting burr (PSD not specified)
Sharma et al. 2020 [16]	United States	Experimental	Two Cadavers	Otologic drill with a 6-mm cutting burr was utilized for each mastoidectomy procedure (Speed not specified)	Fluorescent droplet splatter was visualized via ultraviolet (UV) light	Particle spread detected 91 cm in all directions (PSD not specified)

Of the six studies, five (83%) assessed tools to mitigate particle spread. Four studies assessed the use of a barrier system, with two (33%) using the OtoTent and two (33%) using a barrier drape. Two (33%) studies defined the microscope alone as a possible mitigatory tool. Finally, one (17%) study compared drill burr type and size to determine the effects on particle spread (Table 2). 

**Table 2:** Assessment of the mitigatory tools used to prevent particle spread during mastoidectomy procedure. PSD: particle surface density; SA: surface area.

Author, year	Tool used to mitigate spread (OtoTent, microscope etc.)	Particle spread mitigation
Chari et al. 2021 [11]	OtoTent 1; OtoTent 2	OtoTent 1: 10^4^ µm/L particle density OtoTent 2: 10^1^ µm/L particle density
Chen et al. 2020 [12]	Microscope; Microscope with OtoTent	Microscope with OtoTent: PSD ranged from 0.018 to 0.29 particles/cm^2^ and %SA ranging from 0.008 to 0.25%
Cottrell et al. 2020 [13]	Barrier drape	Barrier drape: Particle spread detected <20 cm from the ear
Lawrence et al. 2020 [14]	Barrier drape	Barrier drape: Visualized contamination to be contained within the tent
Markey et al. 2021 [15]	Microscope; Drill burr	Microscope: Particle spread detected 150 cm in all directions; Drill burr: Using a smaller size cutting burr reduced the spread of contamination; a diamond burr reduced the spread of contamination compared to a cutting burr

Discussion

SARS-CoV-2 poses a significant risk to the surgical team in the operating room due to its marked respiratory transmission [12]. This virus can spread via aerosols, surviving for at least three hours inside, or via fomites, surviving on surfaces such as clinician shoe covers for up to days [13]. Although this article did not specify if the inside space was ventilated or unventilated, the length of time aerosol particles can survive poses a great risk to the medical team, staff and patients. Otolaryngologists are at a higher risk of infection due to their work in and around the airway, particularly in the nasopharynx, as this is the site of SARS-CoV-2 replication [14]. However, there is also a risk of spread during surgical procedures outside of the airway, such as during mastoidectomy, because of the use of drills leading to aerosolization of particles [15]. If surgical teams are not adequately protected from transmission, stability of the healthcare system and its capacity to care for patients may be affected. Because of this concern, our study assessed particle spread during mastoidectomy and ways to mitigate this spread.

At the onset of the COVID-19 pandemic, concern for infection due to particle spread has prompted interest in studying safety protocols during surgical procedures. Particle spread during drilling procedures has begun to be measured in a variety of new ways. In our review, six studies assessed the particle spread during mastoidectomy specifically. Previously, bone scatter and particulate dispersion were assessed; however, as stated in Chari et al., the droplet spread extended much further, ranging from 30 to 208 cm in this analysis [16,17]. While most studies assessed particle spread via fluorescent droplet dispersion, one study [16] assessed particle spread via optical particle size spectrometers. Interestingly, measurement with the optical size spectrometer analysis yielded a maximal distance of 30 cm; however, all studies using fluorescent droplet found spread ranging from 91 to 208 cm, illustrating a possible underestimation of particle spread using the optical size spectrometer. Two studies specifically indicated that there was a spread identified in all directions from the surgical field, pointing to a risk for all surgical team members in the room, regardless of positioning or distance from the patient [17,18].

At the beginning of the COVID-19 pandemic, studies assessed the use of PPE to mitigate particle spread; however, it has been shown that wearing PPE may hinder the wearers’ ability to perform some procedures most effectively. Lawrence et al. found that the use of safety goggles, a full‐face snorkel mask, and full‐face respirator all caused a substantial reduction of the visual field radius by 45%, 60%, and 82% respectively [19]. Lyer et al. noted that simple goggles reduced the visual field radius by 31.6%, with progressive reductions of up to 75.7% with large goggles and 76.8% when a face shield was added [20]. To reduce the hindrance to surgical efficacy associated with PPE worn by the surgical team, while ensuring safety measures to mitigate particle spread are optimized, research on improved protective equipment for mastoidectomy has been conducted.

To date, the most cited protective tool used for mastoidectomy is the OtoTent, which is a barrier drape fashioned from a commercially available drape [19]. Chari et al. analyzed two different OtoTent types, with OtoTent 1 consisting of a drape sheet fit to the microscope and OtoTent 2 consisting of a custom structured drape that encloses the surgical field with specialized ports [16]. In our review, two studies found significant reduction (p<.001, p<.007) in particle density with the use of the OtoTent 2 alone or the OtoTent 1 with a microscope [16,19]. OtoTent 2 showed a significant reduction in particle density, while OtoTent 1 did not. Another study assessed the use of a microscope alone, which reduced particle spread distance by 58 cm [21]. Finally, a generic barrier drape was assessed by two studies in our review; one study found particle spread to be reduced by 180 cm [22] and the other found particle spread to be completely contained within the barrier drape [21]. Interestingly, only one study assessed the benefit of different burr sizes and types, illustrating that using a smaller size cutting or diamond burr reduced the particle spread. Although all of these methods have proven to be beneficial in mitigating spread, the tool which should be used as the standard safety procedure in the operating room remains unknown.

Strengths and limitations

Our systematic review is the first to identify studies that have assessed aerosol and droplet particle spread during mastoidectomy. This review is also the first to analyze potential tools to mitigate particle spread and improve safety for the surgical team. The main limitation of this review is that most studies used fluorescent saline to assess particle spread instead of measuring COVID-19 or other viruses directly. In addition, all but one study used cadaveric bone versus living bone. The use of both fluorescent saline and cadaveric bone could be confounding factors in the measurement of particle spread as well as efficacy of the mitigatory tools. In addition, cauterization may also contribute to the aerosolization of particles during the surgical procedure, and no studies assessed this. This is a possible confounding factor when assessing particle spread mitigation [23]. As well, the collection of aerosolized particles is of key concern in COVID-19 and this requires specialized collection apparatus that have not been employed in these studies [24].

## Conclusions

Particularly in the era of the COVID-19 pandemic, we need to understand how aerosol and droplet particles spread during surgical procedures, including those involving bone cutting such as mastoidectomy. Evaluation of mitigatory tools is imperative for the safety of the surgical team and the healthcare system at large. Barrier drapes, OtoTents and microscopes all have been shown to mitigate particle spread; however, further research needs to be performed to compare their efficacy and develop a standard of safety. Further research should also be performed to assess alternative safety measures, including burr size, speed and type.
